# Cellulose Nanocrystal Allomorphs: Morphology, Self-Assembly, and Polymer End-Tethering toward Chiral Metamaterials

**DOI:** 10.1021/accountsmr.3c00278

**Published:** 2024-03-11

**Authors:** Justin O. Zoppe

**Affiliations:** Universitat Politècnica de Catalunya (UPC) − BarcelonaTech, POLY2 Group, Department of Materials Science & Engineering, School of Industrial, Aerospace and Audiovisual Engineering of Terrassa (ESEIAAT), Carrer de Colom, 11, 08222 Terrassa, Spain

## Introduction

Since the early observations of plane polarized light rotation by tartaric acid by Jean-Baptiste Biot^1^ and Louis Pasteur’s later discovery of tartrate enantiomers in 1848,^2^ the significance of chirality persists throughout natural sciences, technology, engineering, and architecture. We now understand that the building blocks of life, such as nucleic acids, sugars, amino acids, and therefore oligosaccharides and proteins are almost exclusively homochiral.^3^ That is, they lack mirror reflection symmetry and tend to exist in nature with only one handedness, right- (d-isomers) or left-handed (l-isomers). Not 40 years after Pasteur’s work on tartrate crystals, Friedrich Reinitzer and Otto Lehmann discovered what would later be termed cholesteric (chiral nematic) liquid crystals.^4,5^ In the 21st century, liquid crystal displays (LCDs) remain the technology of choice for a wide variety of commercial devices, such as mobile phones, televisions, monitors, instrument panels and signage, although the development of the first operational LCD took nearly 80 years since Reinitzer’s and Lehmann’s first observations. This historical context provides some perspective on where we stand in a new age of liquid crystal research, in which some have turned their attention to *colloids* by virtue of their promise in photonics and metamaterials. In this regard, we have only just begun.

## Colloidal Cholesteric Liquid Crystals

**Figure 1:**
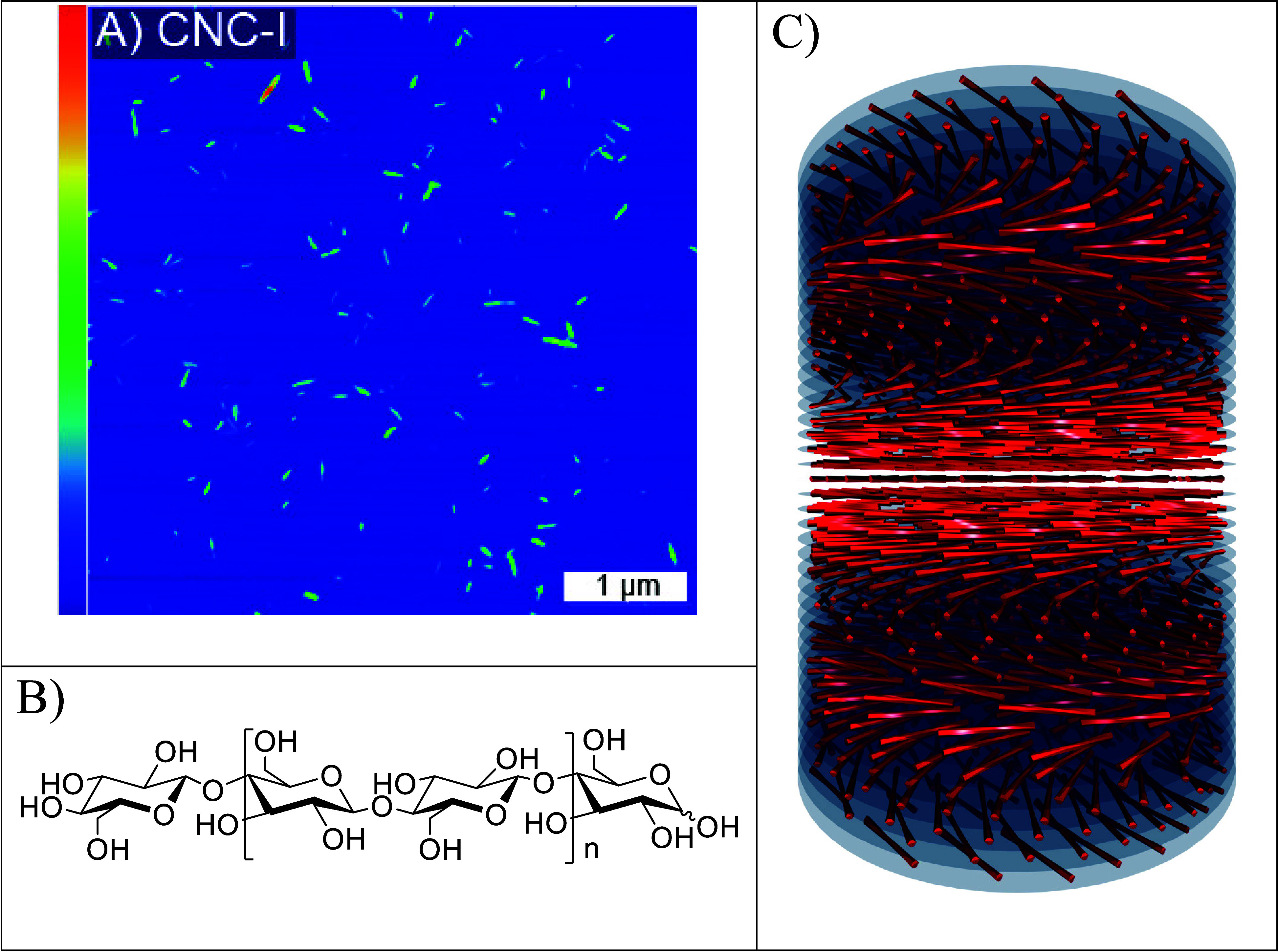
(A) Atomic force microscopy (AFM) height image of rod-like cellulose nanocrystals (CNCs), (B) chemical structure of an individual cellulose chain, consisting of β-1,4-linked β-d-anhydroglucopyranose units with a nonreducing (left) and reducing end (right), and (C) simplified illustration of a left-handed cholesteric liquid crystal domain of CNCs. Reproduced with permission from ref (12). Copyright 2021 American Chemical Society.

Liquid crystals (LCs) are phases of matter that may exhibit fluid-like flow while maintaining a certain degree of anisotropy resembling a solid crystal.^6^ They show either temperature- or concentration-dependent phase transitions between disordered (isotropic) and ordered states, the most common of the latter being nematic, smectic and cholesteric. Cholesteric (N*), also known as chiral nematic or helicoidal, LCs are those in which the nematic director rotates in the form of a helix, which is either right- or left-handed. Their cholesteric pitch (*p*) defines the local helix periodicity within a microdomain. Lyotropic, i.e. concentration-dependent, N* phases are readily observed in colloidal suspensions of rod-like particles, such as filamentous viruses, collagen, chitin, and cellulose nanocrystals (CNCs).^7^ The interest in the cholesteric ordering of CNC dispersions, in particular, has rapidly spread across a variety of disciplines since its discovery just over 30 years ago.^8^ This is due, in part, to the fact that the N* phases of CNC dispersions are further preserved in the solid state upon evaporation-induced self-assembly (EISA), giving rise to photonic films with feature sizes spanning 4 nm to several microns.^7^ CNCs are rod-like, chiral nanoparticles consisting of linear chains of β-1,4-linked β-d-anhydroglucopyranose units with a reducing and nonreducing end (Figure 1A and B). They are produced by selective hydrolysis of the disordered regions of macroscopic semicrystalline cellulose fibers and vary in aspect ratio depending on the source.^9^ The most commonly applied sulfuric acid hydrolysis of cellulose yields CNCs functionalized with anionic sulfate half-ester groups on their surfaces, which provide electrostatic repulsion and a key role in the stabilization of cholesteric phases. Common cellulose sources are vascular plants, fungi, bacteria, algae, and tunicates, producing CNCs with a wide range of average aspect ratios (*L*/*w* = 5 to 200) and elastic moduli (50–220 GPa). The ability of CNCs to form cholesteric solid films upon EISA makes them excellent natural materials for producing films and coatings with structural color^7,10^ and novel materials with mesoporous structures and long-range chiral order, properties useful for many applications in materials engineering.^11^

**Figure 2:**
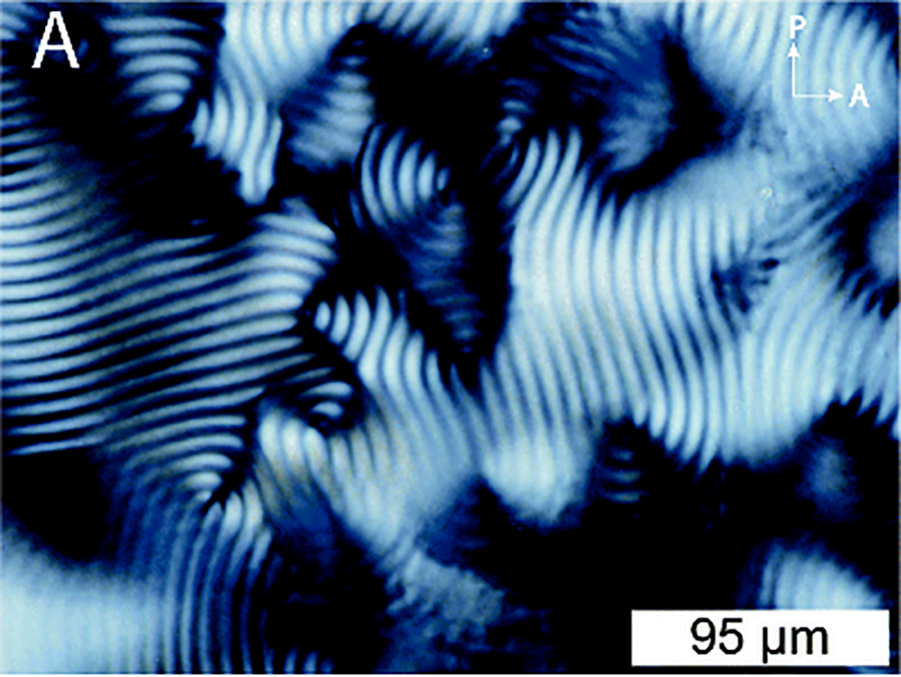
Polarized optical microscopy (POM) image of typical fingerprint texture of the cholesteric phase of aqueous CNC suspensions (5 wt % CNCs, 1 mM NaCl) viewed between crossed linear polarizers. Line spacing corresponds to *p*/2. Reproduced with permission from ref (13). Copyright 2020 Royal Society of Chemistry.

A fundamentally intriguing observation is the fact that CNCs, although individually exhibiting right-handed twists, always form left-handed N* phases (Figure 1C). Right-handed N* phases have, thus far, not been reported. Shown in Figure 2 is a typical fingerprint texture of the left-handed N* phases of unmodified CNC dispersions, in which the line spacing represents half of the cholesteric pitch (*p/2*).^13^ In colloid science, the first theoretical principles that govern isotropic–nematic phase transitions of long hard Brownian rods were developed by Lars Onsager.^14^ According to Onsager’s theory, orientational ordering of ideal rods in the isotropic–nematic phase transition increases translational entropy as a result of a decrease in excluded volume, although at the expense of a loss in rotational entropy of small(er) magnitude. Straley later theoretically predicted the entropy-driven cholesteric phase using threaded rods as model building blocks,^15^ which related the chirality of building blocks to that of their chiral LC phases. In the case of CNCs, their surface charges are expected to be distributed in helical arrangements, due to their right-handed twist, thus inducing a helical rotation of the nematic director.^7^ Simulations suggest that entropy alone can be the sole driving factor in stabilizing cholesteric phases of right-handed building blocks into either left- and right-handed helical arrangements.^16^ The mechanism of chirality transfer in CNC dispersions is still debated, although Chiappini et al. recently proposed that the cholesteric phases of CNCs actually depend on the formation of chiral crystallite bundles, an intermediate stage between individualized CNCs in the isotropic phase and the formation of cholesteric tactoids, the nuclei of LC systems.^17^ The subsequent modeling studies of Sewring and Dijkstra support the hypothesis that chiral crystallite bundles act as chiral dopants of dispersions primarily composed of individual CNCs.^18^ The fact that the individual CNCs were modeled as achiral hard spherocylinders emphasizes the potential importance of chiral amplification in generating a cholesteric phase. As the most widely investigated CNCs present right-handed twists, it would be beneficial to experimentally study phase transitions of achiral cellulose crystallites in order to further test this hypothesis. To this end, our recent work suggests that it is possible to unravel the right-handed twist of CNCs by transforming them into different crystal allomorphs.^13^

In a more general sense, one of the main challenges in colloidal liquid crystal research is precisely controlling the morphological features of nanocolloids, especially chirality, by means of chemical synthesis.^19^ For experimentalists working within the framework of Onsager–Straley theory, there are inherent complications with many rod-like colloidal systems. For example, they are often charge-stabilized, semiflexible or have low aspect ratio, making them deviate from the ideal long hard rods.^14,20^ In order to experimentally address the origin of chirality in cholesteric phases, an experimental platform based on long hard rods not only requires control over the aspect ratio, and dispersity, but also chirality and surface chemistry. CNCs are an ideal class of rod-like colloids to overcome these challenges, since they present (1) aspect ratios up to 200, (2) tunable chirality, and (3) easily adjustable surface chemistry. Contrarily, a more problematic aspect is their length polydispersity. Over the past decade, we have thus started focusing our research efforts on the structure and self-assembly of nanoscale cellulose allomorphs and reducing end-group modification in order to generate new building blocks analogous to both rod–coil diblock and coil–rod–coil triblock copolymers.^12,13,21−24^ Such building blocks can be used as colloid model systems for investigating chirality transfer and show great potential as structure-directing agents for the fabrication of chiral nanostructures.

## Cellulose Nanocrystal Allomorphs

**Figure 3:**
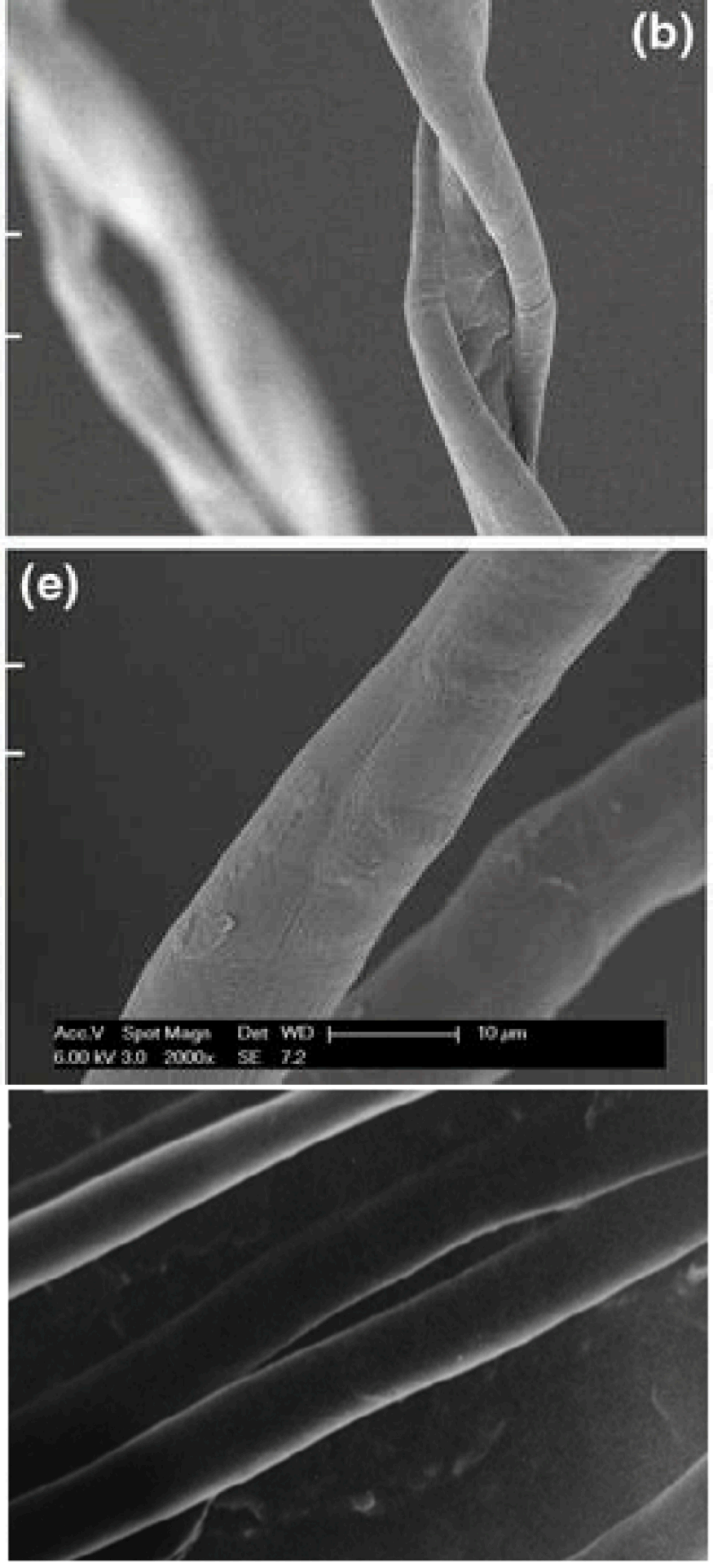
SEM images of (top) untreated- (cellulose I), (middle) mercerized- (cellulose II),^25^ and (bottom) liquid ammonia-treated (cellulose III)^26^ cotton fibers. Reproduced with permission from ref (25). Copyright 2014 Springer Nature. Cellulose III cotton fibers viewed under 640× magnification. Reproduced with permission from ref (26). Copyright 2022 Indian Journal of Fiber & Textile Research.

The native state of the crystalline regions of cellulose fibers is commonly referred to as cellulose I. Depending on the source and treatment of the native cellulose fiber, the hydrogen bonding network and molecular orientation can be reorganized, giving rise to different cellulose allomorphs, namely, cellulose II, III, and IV. The formation of these allomorphs depends on the reagents used in the chemical treatment of the native cellulose I. When native cellulose I is treated with alkali or dissolved in a suitable solvent and regenerated, the allomorph is converted to cellulose II, as in rayon fiber and regenerated cellulose membranes. The structure of cellulose II differs from cellulose I in that some chains are in an antiparallel arrangement where reducing and nonreducing end groups alternate within microfibrils, yielding a more thermodynamically stable structural arrangement. If cellulose I or cellulose II is treated with liquid ammonia or other amines, such as ethylenediamine, followed by swelling agent removal, a so-called cellulose III crystal structure is generated, however differ in the arrangement of reducing end groups depending on the starting material (cellulose I or II). Cellulose III is metastable and reverts back to its original allomorph at elevated temperatures. Upon the aforementioned hydrolysis of macroscopic cellulose allomorphs or posthydrolysis conversion, one can obtain CNCs with crystal structures I, II, or III (CNC-I, CNC-II, CNC-III). Because of the difference in molecular arrangement of cellulose chains within CNC allomorphs (parallel vs antiparallel), the reducing end groups can be located either on one end or both ends.^9^ Moreover, it has been shown that macroscopic cellulose I fibers transformed into cellulose II or cellulose III reveal remarkable changes in fiber twist (Figure 3),^25,26^ which has not been fully elucidated for nanoscale cellulose crystals. We recently showed that cellulose II nanocrystals are also right-handed^13^ and self-assemble into cholesteric phases, however their surface charge distributions and the chirality of cellulose III crystal allomorphs are unknown. As alluded to previously, if they show achiral morphology as in the case of macroscopic fibers, they could provide a system to better understand the role of chirality and crystallite bundles on the formation of cholesteric LC phases by comparison to right-handed cellulose I and II nanocrystals.^18^

## End-Tethered Cellulose Nanocrystal Allomorphs

**Figure 4:**
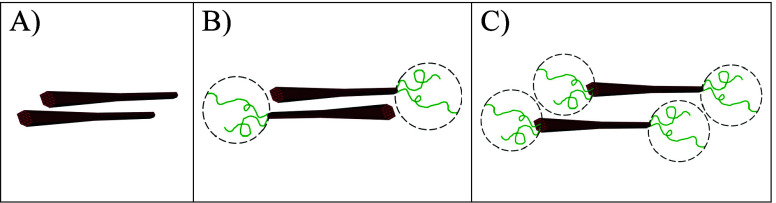
Illustrations of simplified models of (A) a CNC pair, and (B) asymmetric and (C) symmetric end-tethered CNC pairs within a nematic layer of the cholesteric phase. The dotted circles represent volumes occupied by end-tethered polymers. Note that the asymmetric end-tethered CNC pair in (B) is arranged in an antiparallel manner.

The reducing end of cellulose chains reversibly converts between a cyclic hemiacetal and an aldehyde functional group, which provides a unique site for selective end-group chemical functionalization.^22^ As mentioned above, CNC-I have all reducing ends on one extreme, while CNC-II have reducing ends on both extremes. Through grafting of polymers to the end groups of CNC allomorphs, new AB diblock and ABA triblock copolymer nanoparticles are produced.^12,22,23^ Alongside the surface charges of CNCs, end-tethered polymers provide an additional approach to further manipulate the properties of their cholesteric phases due to excluded volume. Shown in Figure 4 are illustrations of a CNC pair, and asymmetric and symmetric end-tethered CNC pairs within a nematic layer of the cholesteric phase, which emphasize some of the proposed effects of the occupied volume of end-tethered polymers on nanoparticle configurations. These arrangements assume that end-tethered polymers are nonadsorbing or depleting, i.e., not attracted to the CNC surface, otherwise the system could form interconnected networks before forming cholesteric phases. Although highly simplified, these illustrations provide some initial insights into the purely morphological contribution of different building blocks on packing within an LC phase. Among the external factors that can be used to manipulate N* phases of CNCs, such as ionic strength, ultrasound, etc.,^9^ surface chemical modification of CNCs with end-tethered polymers remains less explored.^12^ Compared to nonselective surface modification, we hypothesize that selective modification at the ends of nanorods with nonadsorbing polymer tethers allows sufficient packing into N* phases.^12^ In support of this hypothesis, computer simulations have predicted the formation of N* phases of achiral, asymmetric polymer-tethered nanorods within a wide range of their phase diagram.^27^

**Scheme 1:**
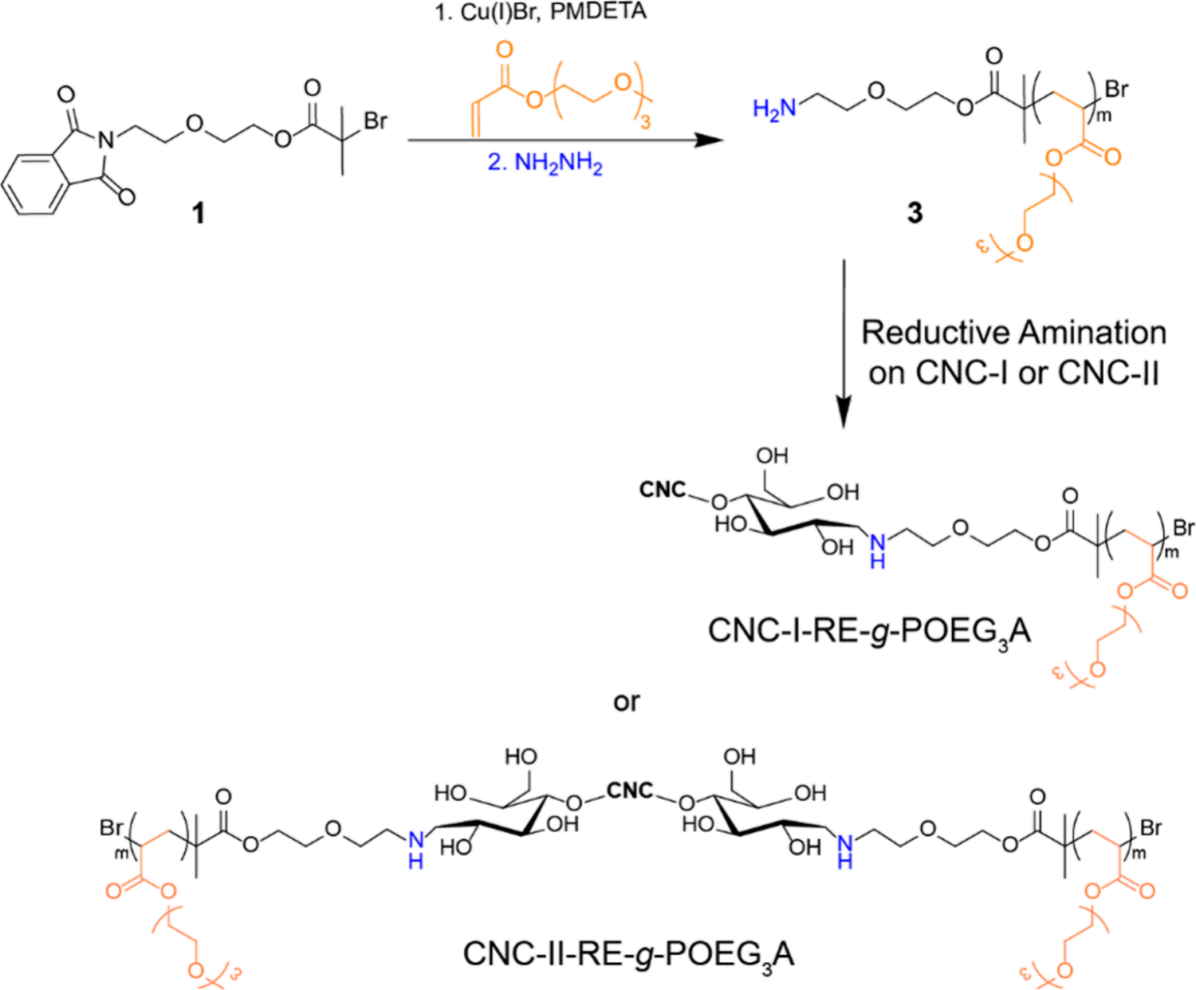
Polymerization of POEG_3_A Using an End-Capped Atom Transfer Radical Polymerization (ATRP)-Initiator, and the Attachment of the Resulting Polymer onto the CNCs via Reductive Amination to Yield CNC-I-RE-*g*-POEG_3_A and CNC-II-RE-*g*-POEG_3_A Reproduced with permission from ref (12). Copyright 2021 American Chemical Society.

In our preliminary work with polyoxyethylene analogues, we found that both asymmetric and symmetric end-tethered CNCs form cholesteric liquid crystal phases.^12^ These hybrid CNCs were prepared by reductive amination of presynthesized poly[2-(2-(2-methoxy ethoxy)ethoxy)ethyl acrylate] (POEG_3_A) at the reducing end groups of cellulose I and II nanocrystals (Scheme 1). POEG_3_A, which was synthesized by atom transfer radical polymerization (ATRP), had a molecular weight of 17 700 g/mol. Interestingly, at a concentration of 10 wt %, chiral nematic tactoids were observed for asymmetric CNC hybrids, which began to merge into larger domains with a pitch of 3.7 μm. In contrast, chiral nematic tactoids appeared for suspensions of symmetric CNCs at a concentration of 14 wt %. Different mechanisms were likely at play, such as the increase in hydrodynamic radius of the particles, the increase in rod length (aspect ratio) and the end-tethered POEG_3_A sterically stabilizes the modified CNCs from both ends, and in turn increases the interlayer separation distance between adjacent CNCs in the cholesteric arrangement, resulting in the large pitch of 6 μm. The end-grafted CNC-II began to self-assemble into cholesteric phases within 1 week, which is about 40 times faster than the case of unmodified CNC-II suspensions.^13^ This could have been a result of a decrease in the rotational diffusion coefficient and/or the depletion interactions caused by end-tethered PEG analogues, potentially reducing the time necessary for orientation into a cholesteric LC phase. As the symmetric CNCs reached a kinetically arrested gel-like state at the concentration of 14 wt %, larger cholesteric domains were not observed over time. From these results, it is clear that end-tethered polymer chemistry, molecular weight and location will have an impact on LC properties, such as the critical concentration, domain size and cholesteric pitch. In order to alter the concentration of kinetic arrest and LC phase characteristics, there are a wide variety of end-tethered nonionic polymers and anionic polyelectrolytes of varying molecular weight to further investigate.

## End-Tethered Cellulose Nanocrystals as Templates for Chiral Metamaterials

One of the potential applications of end-tethered CNCs is directing chiral structures in the fabrication of plasmonic metamaterials by bottom-up self-assembly processes. Chiral metamaterials are emerging media with electromagnetic properties unobserved in nature, such as negative refraction, giant circular dichroism and chiral-selective nonlinear optical phenomena.^28^ Among other applications, they can be used to enhance the inherently weak chiral light–matter interactions, which is essential for sensing and quantifying chiral (macro)molecules.^29^ Examples of chiral metamaterials include inherently chiral plasmonic nanostructures, such as helices and gyroids, or anisotropic achiral plasmonic structures stacked into chiral arrangements. There have been great efforts over the past few years to enhance chiral light–matter interactions using artificial chiral metamaterials fabricated by glancing-angle deposition (GLAD), seed-mediated methods, and block copolymer templating, among others.^30−32^ With respect to chiroptical metamaterials, colloidal liquid crystal templating is a less explored technique, even though their cholesteric phases offer not only inherently helicoidal structures, but also the feature sizes necessary for UV–visible light interactions. To this end, I would argue that end-tethered CNC allomorphs are a most fitting colloidal templating system.

Efforts to form chiral assemblies of metallic nanoparticles or nanorods via coassembly with unmodified CNCs have had limited success in terms of their final chiroptical response, likely due to the sensitivity of the cholesteric phase.^33^ However, end-tethered CNCs in the form of diblock and triblock copolymers could provide new opportunities in the fabrication of chiral metamaterials.^12,22,23^ For example, with an appropriate selection of end-tethered polymers that are known to selectively bind metallic precursors, asymmetric and symmetric end-tethered CNC systems could be used to guide the formation of metallic helicoid metamaterials.^34^ After an appropriate etching process, I envisage such metamaterials templated by cholesteric phases of end-tethered CNCs as an array of Meusnier-type helicoids with morphologies resembling Archimedes’ screws separated by a distance corresponding to the average length of the CNCs employed. Nevertheless, there is a long road ahead and we must first recognize how building block parameters, such as CNC chirality and length dispersity, tether chemistry, nanorod:tether length ratio and symmetry, affect the characteristics of cholesteric liquid crystal phases and films produced via evaporation-induced self-assembly.

## Challenges in Templating

Toward templating of chiral metamaterials, there are many challenges to be addressed in the production of end-tethered CNC building blocks, including producing CNCs with low length dispersity, characterization of nanoscale chirality of various CNC allomorphs, elucidating the distribution of charged surface groups, control of the number of end-tethered polymers and optimization of protocols for templating of metallic nanostructures. The high dispersity of CNCs, especially in terms of rod length, often leads to a defect-rich polydomain structure and nonuniform orientation of helical axes during self-assembly. Size fractionation techniques, such as asymmetric flow field-flow fractionation (AF4), differential centrifugation, centrifugation in a sucrose gradient, and spontaneous cholesteric phase separation, have already shown promising results.^7^ Although more often applied in emulsion purification, osmotic depletion could also be utilized to obtain lower size distributions of CNCs.^35^ In our previous work, we also demonstrated the CNC-II allomorphs, while shorter, have lower length dispersity than CNC-I allomorphs.^13^ However, this characteristic is a double-edged sword since lower aspect ratios likewise alters the thermodynamics of the self-assembly process. With regard to CNC-III allomorphs, this is still an open question and their metastability further complicates their handling (only stable near ambient conditions). Ideally, we would like to produce monodisperse CNC building blocks with aspect ratios exceeding 75, in order to more closely resemble Onsager’s model.^14^ While critical milestones have led to a better understanding of chirality transfer in systems of CNC-I allomorphs,^7,18^ little is known about the effect of polymorphic transformations on the physicochemical properties and self-assembly of CNCs. We wonder how individual CNC chirality, surface chemistry, and end-tethering with nonadsorbing (depleting) polymers affect the formation of crystallite bundles, liquid crystal ordering, and microscopic domain structure.

Based on our recent observations of cholesteric phases from asymmetric and symmetric end group-modified CNCs in aqueous suspensions,^12^ another current challenge is to optimize the EISA process to form solid-state materials with cholesteric order. In a typical EISA of unmodified CNCs, chirality transfer from the individual CNCs to a cholesteric phase is hypothesized to depend on the initial formation of crystallite bundles, followed by the nucleation and growth of tactoids oriented in different directions, which, after kinetic arrest, eventually leads to a defect-rich, mosaic-like polydomain structure.^7,17^ Manipulation of the EISA process through external fields leads to homogeneous photonic films with controllable pitch, producing structural color spanning the visible wavelength range. Cholesteric tactoids of symmetric end-grafted CNCs began to appear at concentrations near the kinetically arrested state; thus, they will not likely form very large cholesteric domains. However, asymmetric CNC hybrids will likely form larger cholesteric domains.^12^ Polyethylene glycols (PEGs) are also well-established depletion agents for a variety of systems, which, in the case of unmodified CNCs, induce attractive interactions and promote rotation, alignment and orientation of tactoids, likely because of excluded volume. Accordingly, we observed that symmetric end-grafted cellulose II nanocrystals assembled into cholesteric tactoids 40 times faster than unmodified cellulose II nanocrystals.^12^ A potential explanation is a combination of a decreased rotational diffusion coefficient and the depletion interactions caused by end-tethered PEG analogues. We suspect that these phenomena will be an advantage in terms of controlling cholesteric domain size and orientation of cholesteric phases of end-tethered CNCs. Here, we wonder how the presence of end-tethered polymers affects the giant permanent electric-dipole moment of CNCs, rotational and translational diffusion coefficients, sedimentation rate and kinetic arrest. Finally, if end-tethered CNCs indeed retain their cholesteric structures during EISA, perhaps moisture annealing,^7^ analogous to annealing of block copolymers for templating of gyroids,^28^ would offer a means to further adjust the properties of cholesteric films.

## Outlook

CNC allomorphs with end-tethered polymer chains show potential not only as a new class of chiral structure-directing agents, but also as unique colloidal models for fundamental studies on the chiral assemblies of condensed matter. It has been 75 years since the development of Onsager’s theory of liquid crystals,^14^ and yet there is still a need for a robust and versatile experimental system of rod-like colloids with tunable chirality and aspect ratio for quantitative testing of his model. Moreover, end-tethered polymers attached to both chiral and achiral CNCs would provide an attractive means to experimentally examine chirality transfer and amplification in cholesteric LC phases. In comparison to current metamaterial fabrication techniques based on vapor deposition, self-assembly processes are unparalleled in terms of cost, processing time and adaptability to large scales. Novel types of helicoidal chiral metamaterials with chiroptical responses in the UV–visible region would open unprecedented possibilities in chemistry, biochemistry, pharmacology, and integrated optical devices for imaging, sensing, and circular polarization.

## Abbreviations

CNC: cellulose nanocrystal
LCD: liquid crystal display
N*: cholesteric or chiral nematic
EISA: evaporation-induced self-assembly
AFM: atomic force microscopy
POEG_3_A: poly[2-(2-(2-methoxy ethoxy)ethoxy)ethyl acrylate]
